# An ambulatory dental treatment of a child with Rett syndrome and limited mouth opening under muscle relaxant-free general anesthesia: a case report

**DOI:** 10.1186/s12871-023-02379-4

**Published:** 2024-01-02

**Authors:** Xiao Tan, Bo Zhu, Yanchen Li, Yuguang Huang

**Affiliations:** 1 Department of Anesthesiology, Peking Union Medical College Hospital, 1# Shuaifuyuan Wangfujing, Dongcheng District, Beijing, 100730 China; 2 Department of Dentistry, Peking Union Medical College Hospital, 1# Shuaifuyuan Wangfujing, Dongcheng District, Beijing, 100730 China

**Keywords:** Rett syndrome, Ambulatory anesthesia, Pediatric anesthesia, Dental treatment, Fast recovery

## Abstract

**Background:**

Rett Syndrome (RTT) is a rare, severe, and progressive developmental disorder with intellectual disability. Anesthesia in RTT patients presents a range of challenges. We report a child with RTT who received dental treatment under muscle relaxant-free general anesthesia in our ambulatory center.

**Case presentation:**

A 15-year-old girl with RTT was admitted to our dental clinic with multiple dental caries and residual roots. Dental treatment was scheduled under ambulatory general anesthesia. After anesthesia induction, a nasal tube was initiated under the guidance of a fiberoptic bronchoscope. Multimodal analgesia, body temperature monitoring, and postoperative nausea and vomiting prevention were applied. No muscle relaxants were used throughout the process. The endotracheal tube was successfully removed after the operation and the patient was discharged home the same day.

**Conclusion:**

An individualized anesthesia strategy enabled a quick and safe recovery for this RTT patient after dental treatment under muscle relaxant-free general anesthesia.

**Supplementary Information:**

The online version contains supplementary material available at 10.1186/s12871-023-02379-4.

## Background

Rett Syndrome (RTT) is a severe, rare, and progressive developmental disorder with intellectual disability [1]. Mutations of the methyl-CpG binding protein 2 (MECP2) gene on the X chromosome are the most prevalent cause of classical RTT cases [2]. Characteristic symptoms of RTT include partial or complete loss of acquired spoken language and motor skills, repetitive hand movements, breathing irregularities and seizures. RTT patients may also suffer from scoliosis, wasting, dystonia and bradykinesia. Many patients are wheelchair and/or gastrostomy-tube dependent in their motor deterioration stage [3]. We report here a child with Rett syndrome who received dental treatment under muscle relaxant-free general anesthesia (GA) in our ambulatory center.

## Case presentation

A 15-year-old girl (height approximately 150 cm and weight 37 kg, body mass index 16.4 kg/m^2^, with muscle weakness and wheelchair dependence) was admitted to our dental clinic with chief complaints of multiple dental caries and residual roots. Due to her intellectual disability and poor tolerance to dental examination and invasive treatment, ambulatory dental surgery was scheduled under general anesthesia. The patient presented with symptoms of growth retardation, scoliosis, inability to stand up, and intellectual disability at one year of age and was diagnosed with Rett syndrome in another hospital thereafter. The patient also had epilepsy which was well controlled on valproate and levetiracetam. Preoperative evaluation was performed in the anesthesiology outpatient department. The patient had a difficult airway due to limited movement of the temporomandibular joints. A Mallampati score of IV and limited mouth opening of 1 finger breadth were detected.

The patient was kept nil per os (NPO) for more than 6 h before surgery and received no preoperative sedative. In addition to standard monitoring by the American Society of Anesthesiologists, bispectral index (BIS) monitoring was initiated before anesthesia induction. Sevoflurane 6% in 100% oxygen at 6 L/min was inhaled to facilitate the placement of an intravenous catheter. Total intravenous anesthesia (TIVA) was conducted after the surgery. No difficult mask ventilation was found during preoperative evaluation and sevoflurane inhalation. Anesthesia was induced with target-controlled infusion (TCI) of propofol and intravenous injection of remifentanil 1 mcg/kg. Neuromuscular blockade (NMBA) was not administered. A cuffed 6.0 mm internal diameter nasal Ring-Adair-Elwyn (RAE) tube was safely and smoothly intubated under the guidance of a fiberoptic bronchoscope. Body temperature was maintained by administering prewarmed intravenous fluid and a forced-air warming blanket, and nasopharyngeal temperature was continuously monitored after the induction of general anesthesia until the end of surgery, increasing from 35.8 ℃ to 36.3 ℃. NMBA-free total intravenous anesthesia with propofol TCI and remifentanil infusion was continuously administered to maintain the BIS value between 50 and 60. Fentanyl was added intermittently according to surgical stimulation, vital signs and BIS monitoring, with a total dosage of 75 mcg.

The dental procedure performed in this case involved the use of gutta-percha and resin for filling cavities, as well as extraction of remaining roots. Local anesthetics were administered for pain relief. The entire procedure lasted 4.5 h, during which 600 ml of fluid was infused without placement of a urinary catheter. Towards the end of the procedure, flurbiprofen 50 mg was administered for postoperative analgesia. Ondansetron 4 mg and dexamethasone 3 mg were given intravenously for the prevention of postoperative nausea and vomiting (PONV). Spontaneous breathing recovered within 10 min after discontinuation of propofol and remifentanil infusion. After the safe removal of the RAE tube, the patient was transferred to the post-anesthesia care unit (PACU), where she was accompanied by her parents. She stayed in the PACU for 1 h before being discharged home. The postoperative recovery was uneventful.

## Discussion and conclusions

RTT is recognized as one of the primary causes of genetic intellectual disability and developmental regression in females [4]. Anesthesia management in RTT patients is primarily documented through a limited number of case reports. The drivers for dental intervention in RTT include bruxism and dental caries [5]. Dental surgery in RTT children presents a range of challenges to anesthesiologists [6], including managing an uncooperative child with social disability, addressing difficult airway management, and ensuring rapid recovery from ambulatory procedures.

To our knowledge, this is the first report of RTT patient undergoing ambulatory surgery under general anesthesia. The case reported by Wisam et al. was discharged on the second day following dental treatment [7], while the case reported by Ponde et al. was transferred to the pediatric intensive care unit (PICU) for postoperative monitoring [8]. In our institute, it is routine clinical practice for patients receiving dental treatment under general anesthesia to be discharged on the same day. For this child with RTT who was completely care-dependent, efforts were made to ensure the patient readiness for discharge after ambulatory anesthesia. Discharge assessment was based on stable vital signs, control of pain and PONV, and ensuring that the patient was able to stand and walk independently [9–11]. Rapid discharge could not jeopardize safety. RTT patients tend to be highly sensitive to anesthetic agents and muscle relaxants, and delayed recovery has been reported [12]. Therefore, day-case surgery anesthesia in RTT patients is both noteworthy and challenging. An individualized anesthesia approach was adopted, including the use of short-acting anesthetics, reducing the type and dose of drugs, preventing PONV, and multimodal analgesia. The patient was discharged safely on the day of surgery, with a pediatric post-anesthetic discharge scoring system (Ped-PADSS) score of 9 [13]. One point was deducted because the patient was unable to walk independently, which was consistent with her preoperative condition.

Airway management for an uncooperative patient with limited mouth opening poses a significant challenge to anesthesiologists. Adequate preoperative airway assessment is essential, but it is often difficult in individuals with intellectual disabilities and autistic behavior [14]. In this patient, following a meticulous assessment in the anesthesia preoperative evaluation clinic (APEC) and immediately before induction of anesthesia, we successfully performed FOB-guided nasal RAE endotracheal intubation under the premise of proper mask ventilation. As the patient had experienced muscle wasting, we did not administer NMBA. Ponde et al. [8] also employed an NMBA-free and opioid-free strategy in an RTT patient undergoing dental procedures. Notably, the use of NMBA is not an absolute contraindication in RTT patients. Nho et al. [14] reported a case of an RTT patient with limited mouth opening who received NMBA for FOB-guided endotracheal intubation. In cases of RTT with difficult airway predictors, as reported by Motomura et al. and España et al., NMBA was also used before intubation [15, 16]. Based on our clinical experience, a strategy of using opioids in combination with an NMBA-free approach is a feasible way to perform nasal intubation and dental treatment, with the potential benefit of avoiding NMBA-related complications in patients with muscle weakness.

BIS was applied as anesthesia monitoring in this case. The application of BIS in patients with a history of epilepsy is worthy of discussion. As an EEG-derived variable, any clinical conditions that affects brain electrical activity, including epilepsy, have a potential impact on BIS. Fortunately, epilepsy was well controlled in this patient, so we believe the employment of BIS in this case have certain reference significance. Kimura et al. and Kawasaki et al. describe BIS application of patients with RTT undergoing general anesthesia [17, 18]. They believe that BIS may be useful for patients with RTT. Kim et al. monitored BIS in a patient with RTT and poor control of epilepsy during scoliosis surgery [19]. Although the patient experienced delayed emergence, the author discussed that it might be ideal to maintain BIS at around 50, rather than the lower limit (30–45). Dahaba et al. studied the relationship between BIS and electrocorticographic spikes in patients receiving epilepsy surgery [20]. In their study, BIS well reflected the depth of anesthesia, either under sevoflurane or propofol anesthesia. In summary, we believe that as an increasingly used monitoring in recent years, application of BIS facilitates anesthesiologists in attaining enhanced safety during anesthesia. However, BIS cannot be considered a true reflection of depth of anesthesia [21], and the control of anesthesia depth is contingent upon the anesthesiologist’s clinical expertise and comprehensive discernment.

Perioperative hypothermia is associated with adverse clinical outcomes [22]. Temperature abnormalities are commonly described in clinical reports of patients with RTT [23]. In this case, the patient was wearing thick clothing upon entry into the operating room. Based on the hypothermia prediction model proposed by Yi et al. [24], she was classified as a patient at higher risk for intraoperative hypothermia. It was recommended to use forced air warming blankets before and during the surgery. In this case, temperature monitoring was performed intraoperatively. The use of pre-warmed intravenous fluids and forced air warming blankets was shown to be effective in preventing hypothermia and promoting rapid recovery after surgery.

Other anesthesia considerations for RTT patients include epilepsy control [15], electrocardiogram abnormalities [6], and respiratory complications [25–27], etc. However, in this patient, the relevant systems are generally stable or well-controlled, and thus will not be further discussed.

Individualized anesthesia strategy is recommended for patient with RTT, according to different disease profile and surgery types. In this case, we describe the anesthesia management of an RTT patient undergoing ambulatory dental treatment. With multimodal analgesia with avoidance of NMBA, airway management strategy and temperature maintenance, we ensured patient safety and fast recovery without the need for hospitalization.

### Electronic Supplementary Material

Below is the link to the electronic supplementary material.

**Supplementary Material 1:** CARE Checklist

## Data Availability

The datasets and clinical data used and/or reported in the current study are available from the corresponding author on reasonable request.

## Abbreviations

APEC: anesthesia preoperative evaluation clinic
BIS: bispectral index
GA: general anesthesia
MECP2: methyl-CpG binding protein 2
NPO: nil per os
NMBA: neuromuscular blockade
PICU: pediatric intensive care unit
Ped-PADSS: pediatric post-anesthetic discharge scoring system
PONV: postoperative nausea and vomiting
PACU: post-anesthesia care unit
RTT: Rett Syndrome
RAE: Ring-Adair-Elwyn
TCI: target-controlled infusion
TIVA: total intravenous anesthesia
